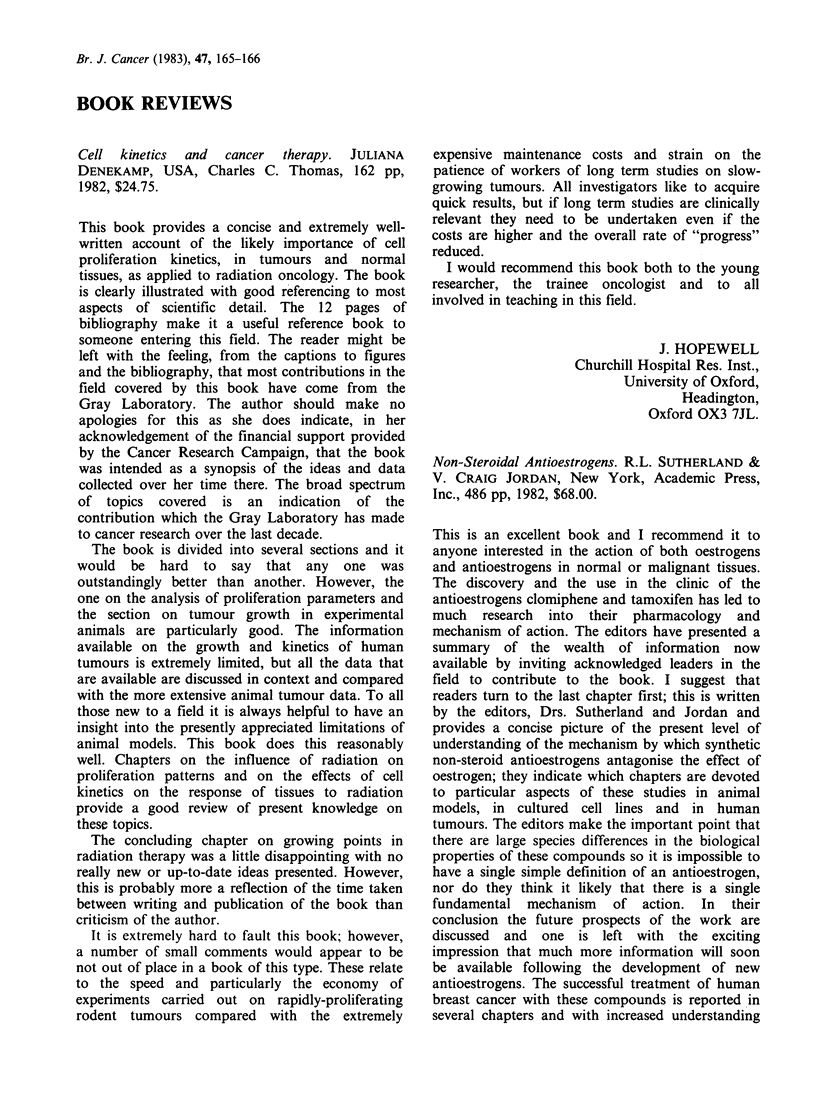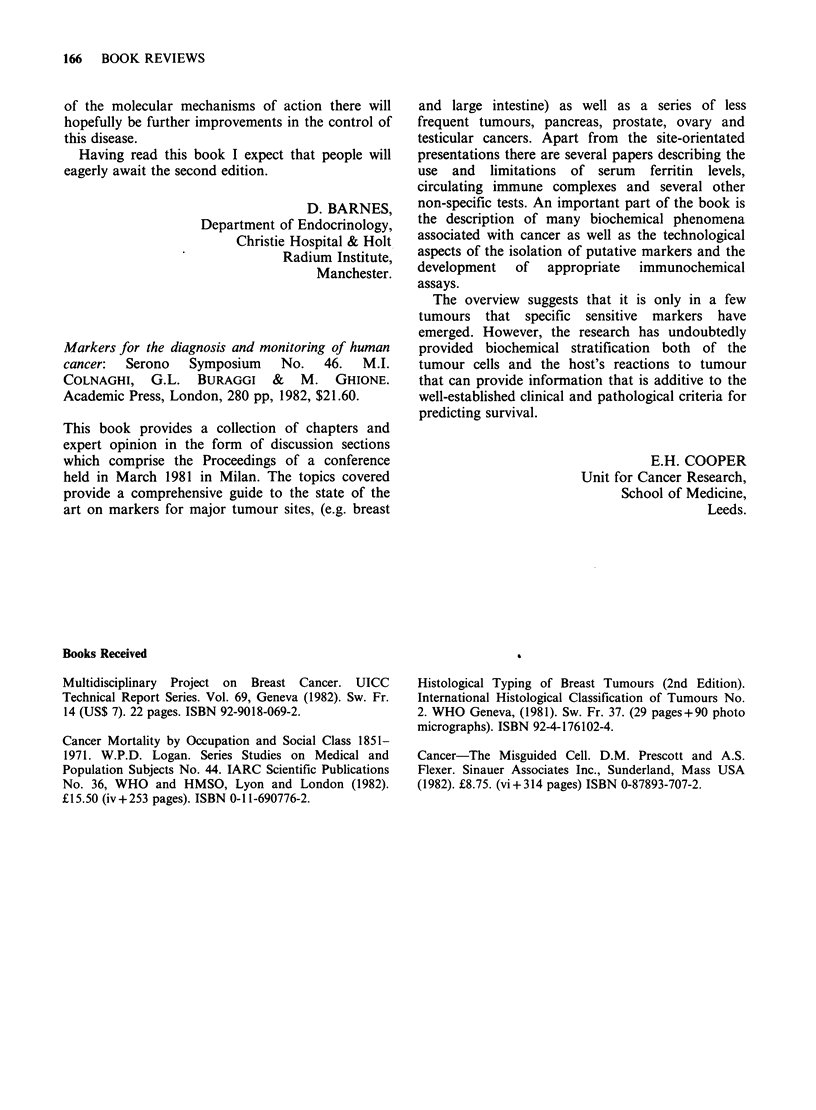# Non-Steroidal Antioestrogens

**Published:** 1983-01

**Authors:** D. Barnes


*Non-Steroidal Antioestrogens*. R.L. SUTHERLAND & V. CRAIG JORDAN, New York, Academic Press, Inc., 486 pp, 1982, $68.00.

This is an excellent book and I recommend it to anyone interested in the action of both oestrogens and antioestrogens in normal or malignant tissues. The discovery and the use in the clinic of the antioestrogens clomiphene and tamoxifen has led to much research into their pharmacology and mechanism of action. The editors have presented a summary of the wealth of information now available by inviting acknowledged leaders in the field to contribute to the book. I suggest that readers turn to the last chapter first; this is written by the editors, Drs. Sutherland and Jordan and provides a concise picture of the present level of understanding of the mechanism by which synthetic non-steroid antioestrogens antagonise the effect of oestrogen; they indicate which chapters are devoted to particular aspects of these studies in animal models, in cultured cell lines and in human tumours. The editors make the important point that there are large species differences in the biological properties of these compounds so it is impossible to have a single simple definition of an antioestrogen, nor do they think it likely that there is a single fundamental mechanism of action. In their conclusion the future prospects of the work are discussed and one is left with the exciting impression that much more information will soon be available following the development of new antioestrogens. The successful treatment of human breast cancer with these compounds is reported in several chapters and with increased understanding of the molecular mechanisms of action there will hopefully be further improvements in the control of this disease.

Having read this book I expect that people will eagerly await the second edition.

D. BARNES,
Department of Endocrinology,
Christie Hospital & Holt
Radium Institute,
Manchester.